# Building an interpretable machine learning prognosis prediction model-based on baseline examinations of patients with esophageal cancer undergoing surgery

**DOI:** 10.3389/fonc.2026.1839382

**Published:** 2026-08-14

**Authors:** Ruiyu Zhan, Qifeng Wang

**Affiliations:** 1 Department of Oncology, Zigong Fourth People’s Hospital, Zigong, China; 2 Department of Radiation Oncology, Affiliated Cancer Hospital of University of Electronic Science and Technology of China, Chengdu, China

**Keywords:** esophageal cancer, machine learning, prognosis, Shap, WSO

## Abstract

To create an interpretable machine learning model based on non-invasive biomarkers for the early diagnosis and improved prognostic value of esophageal cancer. We gathered a private dataset at Sichuan Cancer Hospital, comprising 3204 esophageal cancer patients who underwent surgery. Baseline markers and preoperative biochemical blood tests were thoroughly reviewed. The necessary factors were identified using an elastic net, and 27 machine learning methods were used to construct prediction models. The random forest model was chosen for its high performance, and additional optimization was performed using the White Shark Optimizer (WSO) algorithm. To improve the model’s interpretability, the Shapley Additive exPlanations (SHAP) technique was used. We proposed an interpretable machine learning framework for exploratory early risk stratification of postoperative esophageal squamous cell carcinoma patients using routinely available preoperative baseline examinations. The random forest (RF) model achieved 0.74 accuracy and 0.72 F1 score after 1 year. It was adjusted to 0.69 in the Area Under the Curve (AUC). Gender and blood magnesium levels were the best six characteristics that affected the 1-year and 5-year survival rates. The model with the highest discriminative ability accurately predicted the prognosis of esophageal cancer patients in the test population. Our research led to the creation of a machine learning model that accurately predicts the prognosis of esophageal cancer and identifies early indicators of adverse outcomes. SHAP-improved interpretability facilitates physician-patient interaction and trust and promotes individualized therapy.

## 1 Introduction

Cancer-related statistics all over the world in 2022 reveal that esophageal cancer is the seventh most frequent cause of death in the sphere of cancer-related disease and is known to be fatal and thus spreading aggressively (1). Although the AJCC TNM staging system has been widely applied, the inclusion of additional features can effectively analyze the patient’s condition, formulate treatment plans, and assess disease progression, thereby helping to develop specific treatment plans (2). The important case is early intervention with high-risk patients (2). Nevertheless, even in the face of the aforementioned abundance of biomarkers in baseline checks, the range of useful instruments that can be applied on a broad scale in clinical routine remains significantly limited (3). This highlights the urgent need to establish clinical prognostic models that utilize baseline assessment information and have the potential to detect and identify new prognostic biomarkers and formulate integrated predictive models (4). It is projected that big data should be utilized to leverage laboratory indicators, in conjunction with survival outcomes, to inform valuable clinical decisions (4).

Conventional statistical tools face several limitations in building prediction models for esophageal squamous cell carcinoma (ESCC). These limitations include limited sample size, limited range in the evaluation of predictive variables, and reproducibility issues, among others, that thwart their use in clinical practice (2, 3). On the contrary, machine learning (ML) holds great promise for overcoming these challenges. The ability of ML to manipulate multidimensional variables and reveal complex, nonlinear correlations between clinicopathological variables and outcomes is inherently beneficial (1–4).

Although ML has impressive potential for diagnosing esophageal cancer (5) and predicting prognosis (4, 5), its black-box nature is a serious barrier to its widespread use in clinical settings (6). Modern prognostic models, especially for esophageal cancer, tend to prioritize accuracy over transparency (7, 8), thereby restricting their application in clinical practice (9). To address this issue, explainable artificial intelligence has attracted significant attention. The intended results of these approaches are to understand better how they made their decisions (10, 11). To exemplify, the SHAP approach has proven to be successful in other medical fields (12–14) but not in the Area of prognosis prediction of esophageal cancer based on baseline assessment data.

To fill this research gap, we proposed an interpretable machine learning framework for exploratory early preoperative risk stratification in patients with esophageal squamous cell carcinoma undergoing surgery. We carefully selected the baseline examination markers, patient age, and physical status score as model inputs, and used advanced ML algorithms for training and optimization. The objective of this study is to develop and interpret an exploratory machine learning model for early preoperative risk stratification using routinely available clinical indicators. The proposed model is intended to provide supportive information for clinical decision-making and future individualized perioperative management. The code is available: https://github.com/ruiyuzhan/IML_esophageal_cancer_survival.

## 2 Methods

**Figure 1:**
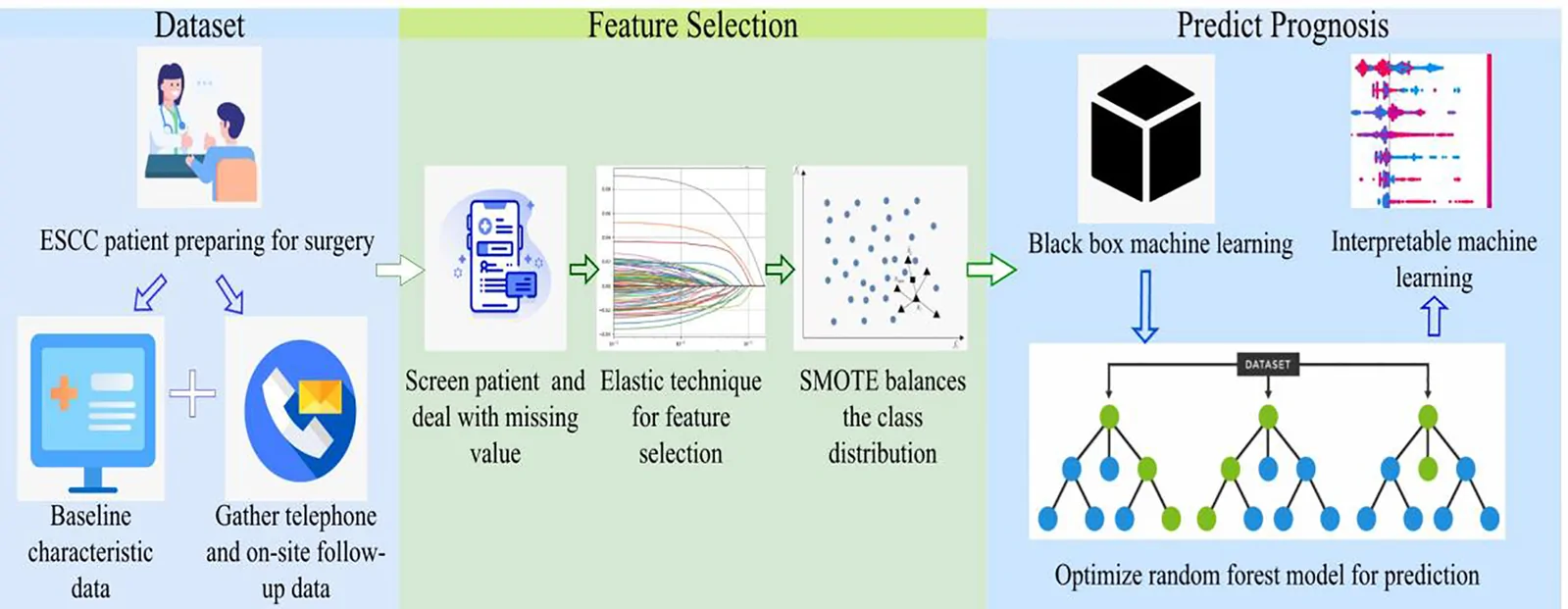
The suggested general plan for the paper consists of three main steps: dataset preparation, feature selection, and prognosis prediction.

This section mainly outlines the research subjects and methodologies used, and the research flow is reflected in the diagram below. The general proposed pipeline is presented in **Figure 1**.

### 2.1 Dataset

This retrospective study included patients who were histopathologically diagnosed with esophageal squamous cell carcinoma (ESCC) and underwent surgery at Sichuan Cancer Hospital between 2009 and 2023. The general study workflow is shown in **Figure 1**.

The inclusion criteria were as follows: (1) histopathological confirmation of ESCC without distant metastasis; (2) tumor location outside the cervical esophagus; (3) no previous history of cancer-specific treatment; and (4) availability of complete clinical, hematological, and follow-up records. The exclusion criteria were as follows: (1) history of other malignancies or perioperative death; (2) cervical esophageal involvement; (3) insufficient follow-up information; and (4) follow-up duration of less than 6 months.

**Figure 2:**
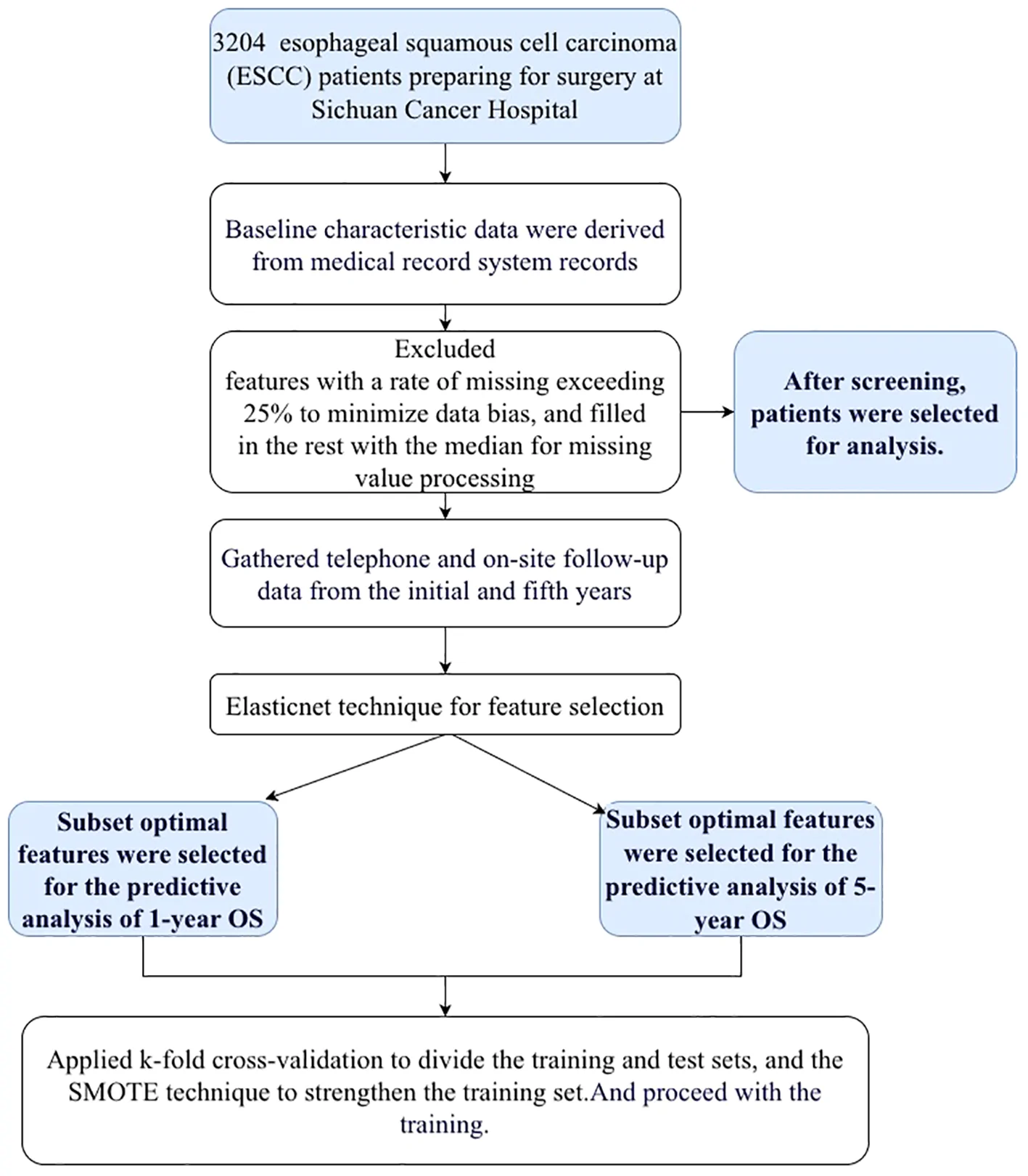
Flow chart of data set collection.

After applying the above eligibility criteria and reviewing the medical records, 3,204 surgically treated ESCC patients were initially identified. To derive the final analytic cohort, variables with more than 25% missing values and patients with more than 25% missing baseline features were excluded. After this missing-data filtering step, 2,352 patients remained and were included in the final analysis. The reduction from 3,204 to 2,352 patients (n = 852) resulted entirely from exclusion of patients with >25% missing baseline features. The cohort-selection process is summarized in **Figure 2**.

The same final analytic cohort of 2,352 patients was used for both endpoint-specific analyses. For the 1-year overall survival (OS) endpoint, 556 patients (23.6%) died within 1 year and 1,796 patients (76.4%) survived beyond 1 year. For the 5-year OS endpoint, 1,553 patients (66.0%) died within 5 years and 799 patients (34.0%) survived beyond 5 years.

The study protocol was approved by the Ethics Committee of Sichuan Cancer Hospital SCCHEC-02-2020–015 and was conducted in accordance with the Declaration of Helsinki. Written informed consent was obtained from all patients.

In this paper, all patients in the sample underwent detailed medical history, past medical history examination, and a comprehensive basic examination, including KPS scores, imaging, hematology, and biochemical indicators (presented in **Supplementary Table 1**).

### 2.2 Feature selection

Data on baseline characteristics were obtained from the medical record system during the first week of patient admission to identify features and construct a predictive model. The use of the Elasticnet method for feature selection in our survival prediction model (**Figures 1**, **2**) has enabled us to leverage the utility of both ridge and lasso regression as penalized regression methods (15).

We dropped the smoking and alcohol consumption features because of missing values above 25. Meanwhile, such characteristics as the history of hypertension and diabetes in the past were eliminated as well. We omitted patients who had above 25% features absent at the time of collection. Of the screened patients, 2352 were left for further analysis. To handle missing values, we used the median to impute missing values. The prediction model was developed using the selected features. We use Z-score scaling in our methodology.

We collected telephone and on-site follow-up information in the first and fifth years to comprehensively compare the short and long-term outcomes of patients, i.e. overall survival. Subsequently, the selected features were used to select feature sets for 1-year and 5-year survival rates, comprising numerous dimensions, including age, gender, KPS score, cardiovascular disease history, imaging, hematological indicators, and biochemical indicators. The correlation map of the chosen features used in the 1-year prediction is presented in **Supplementary Figure 1**, and the correlation map of the selected features required in the 5-year prediction is presented in **Supplementary Figure 2**.

### 2.3 Predict prognosis

#### 2.3.1 Preprocessing pipeline

After cohort-level inclusion/exclusion criteria and missingness filtering, the final analytic cohort was used for model construction. ElasticNet-based feature selection was performed during the model-development stage using cross-validation for regularization-parameter tuning. For algorithm comparison, stratified k-fold cross-validation was performed. In each cross-validation iteration, the data were first split into a training fold and a held-out evaluation fold. The held-out evaluation fold was not used for feature selection, imputation, scaling, SMOTE, or model training.

Within each fold, median imputation was fitted using the training fold only and then applied to the corresponding held-out fold. Continuous variables were subsequently standardized using Z-score normalization, with the mean and standard deviation estimated from the training fold only. The same scaling parameters were then applied to the held-out fold.

Because of class imbalance in the outcome distribution, the Synthetic Minority Oversampling Technique (SMOTE) was applied to the training data. Importantly, SMOTE was performed only on the training fold after median imputation and Z-score normalization. The held-out fold was not resampled and was used only for model evaluation. Therefore, no information from the held-out fold was used during imputation, scaling, SMOTE, or model training. The basic idea of SMOTE is to expand the data set by creating artificial samples in the attribute space, based on a specific instance with its K-nearest neighbors (16). This helps prevent overfitting and enables the classifier to determine the decision boundaries between classes.

#### 2.3.2 Machine learning algorithms

Model development began with a benchmark comparison of 27 machine learning algorithms to evaluate their prognostic performance for 1-year and 5-year OS prediction. Specifically, the evaluated algorithms included adaptive boosting, bagging, Bernoulli naïve Bayes, calibrated classifier with cross-validation, decision tree, dummy baseline classifier, extremely randomized tree, extremely randomized trees, Gaussian naïve Bayes, k-nearest neighbors, label propagation, label spreading, linear discriminant analysis, linear support vector machine, logistic regression, nearest centroid classifier, Nu-support vector machine, passive-aggressive classifier, perceptron, quadratic discriminant analysis, random forest, ridge classifier, cross-validated ridge classifier, stochastic gradient descent classifier, support vector machine, extreme gradient boosting, and light gradient boosting machine. All candidate algorithms were assessed under the same preprocessing and cross-validation framework. The detailed settings of these algorithms are provided in **Supplementary Table 2**. Based on the benchmarking results, the best-performing model was selected for further hyperparameter optimization.

#### 2.3.3 Evaluation metrics

We created a prediction model based on the selected features and evaluated its reliability using various assessment metrics, including the Area under the ROC curve (AUC), F1 score, and accuracy.

#### 2.3.4 SHAP

We used the SHAP methodology to enhance the interpretability and meaning of our model. Lundberg and Lee proposed SHAP in 2017 (13, 16), a technique of individual prediction explanation. Not only can SHAP measure the significance of each input feature, but it can offer both global and local explanations (13, 16), to enable clinicians and patients to intuitively interpret which features have the most contribution to prognostic prediction and their mechanism of action, which makes black box models ineffective.

#### 2.3.5 Implementation details

The tools used in the present research included MATLAB 2024a, Anaconda 2023, and Python 3.10 for data processing and model development.

## 3 Experiment results

### 3.1 Classification results

**Figure 3:**
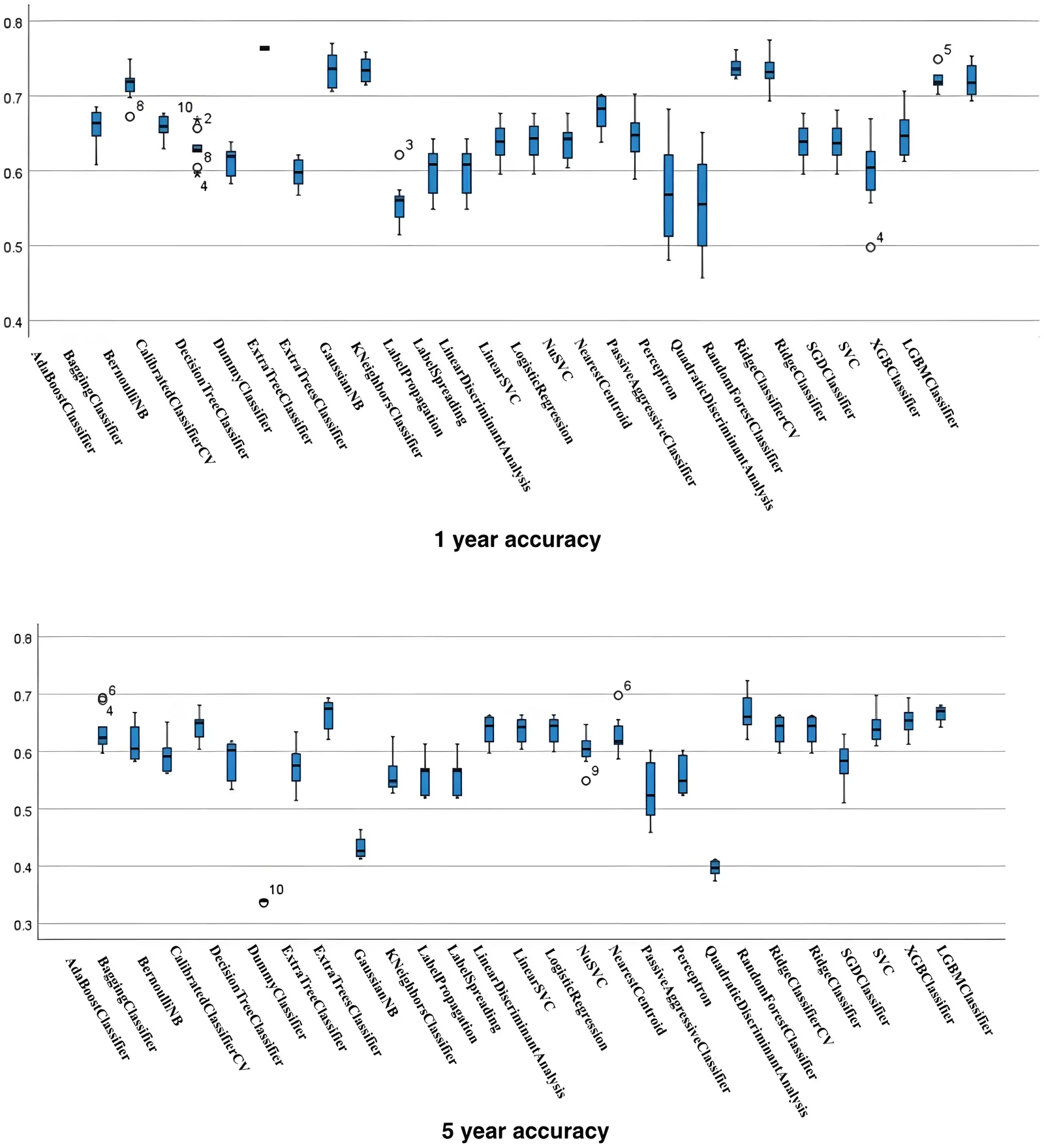
Performance model in terms of 1-year and 5-year prediction accuracy. The images in the boxplots show the number of outliers.

We modelled 27 machine learning models to predict patient prognosis based on the admitted baseline examination data of patients with esophageal cancer. Cross-validation (10-fold) was performed. The random forest model was the most effective in predicting 1-year survival, with an accuracy of 0.74, a satisfactory AUC of 0.69, and an average F1 score of 0.72. The next model was the XGBoost Classifier, which also performed well but was marginally lower than the RF model across many measures. **Figure 3** presents the discriminative predictive performance of these 27 models in a 1-year survival graph. We have analyzed the relationships among the chosen features for predicting 1-year and 5-year survival.

When switching to 5-year survival prediction, with the augmented level of difficulty in the task, the RF model retained its first place with high accuracy (average = 0.67), AUC (average = 0.62), and F1 score (average = 0.67). It is notable that the Logistic Regression model also performed well in predicting 5 years out, with accuracies of 0.64, 0.64, and 0.65, respectively, and came second. **Figure 3** provides a general overview of the discriminative performance of these models in predicting 5-year survival. The Random Forest algorithm (RF) demonstrated the best predictive ability for esophageal carcinoma outcome. The metrics applied to assess model performance were AUC and F1-score. The AUC results for 1-year and 5-year predictions are shown in **Supplementary Figure 3**, and the F1-score results are shown in **Supplementary Figure 4**. Also, **Supplementary Figure 5** shows the RF model’s confusion matrix results.

We have critically examined the preoperative biochemical blood tests and baseline markers to build and verify a clear, interpretable machine learning model to foresee the results of esophageal cancer. This is done to improve prognostic accuracy and understanding, which sets it apart from other research projects. **Table 1** presents a detailed comparison of our optimal Random Forest (RF) model with SMOTE and WSO against state-of-the-art (SOTA) published models used for prognosis prediction. It is also important to note that our paper uses a larger sample of 3204 preoperative patients with non-invasive biomarkers.

**Table 1:** Comparison of proposed techniques and earlier techniques of survival prediction in esophageal cancer.

References	Dataset	Classifiers	Accuracy	F1 score	AUC
Beukinga et al., 2022 (17)	Clinical data (199 patients)	SVM, NB, KNN, RF,NN	0.66	N/A	0.67
Nopour et al., 2024 (18)	Clinical data (3256 patients)	RF, SVM, XGBoost	0.87	N/A	0.83
Hu et al., 2021 (19)	Image data (231 patients)	SVM	0.771	N/A	0.805
Ours	Preoperative non-invasive biomarkers data (3204 patients)	RF (1year) RF (5 year)	0.74 0.67	0.72 0.62	0.69 0.67

### 3.2 In-depth modelling optimization

The proposed research is expected to significantly improve model performance by optimizing the RF model in depth through the state-of-the-art White Shark Optimization algorithm. The WSO algorithm is based on the biological behavior of white sharks in the deep sea, which use their outstanding auditory and olfactory senses to follow, find, and hunt prey (20). With the emphasis on the main parameters of the algorithm used- tree_num, minleafsize, and NumPredictors - when optimizing the model, it is important to pay a lot of attention to their fine-tuning, which can help to improve the performance of the model.

WSO provided an automated and reproducible strategy for hyperparameter search. To evaluate its incremental benefit, we compared the WSO-optimized random forest with both the default random forest and grid-search-optimized random forest under the same stratified 10-fold cross-validation setting. For 1-year OS, the mean AUC increased from 0.614 with the default random forest to 0.622 after WSO optimization, whereas grid search achieved a mean AUC of 0.620. For 5-year OS, the mean AUC increased slightly from 0.626 with the default random forest to 0.627 after WSO optimization, whereas grid search achieved a mean AUC of 0.625. These results show that WSO produced only a small improvement over the default random forest and grid search. Therefore, we interpreted WSO mainly as an automated and reproducible hyperparameter-search method, rather than as a method that substantially improved discrimination performance.

### 3.3 SHAP results analysis

**Figure 4:**
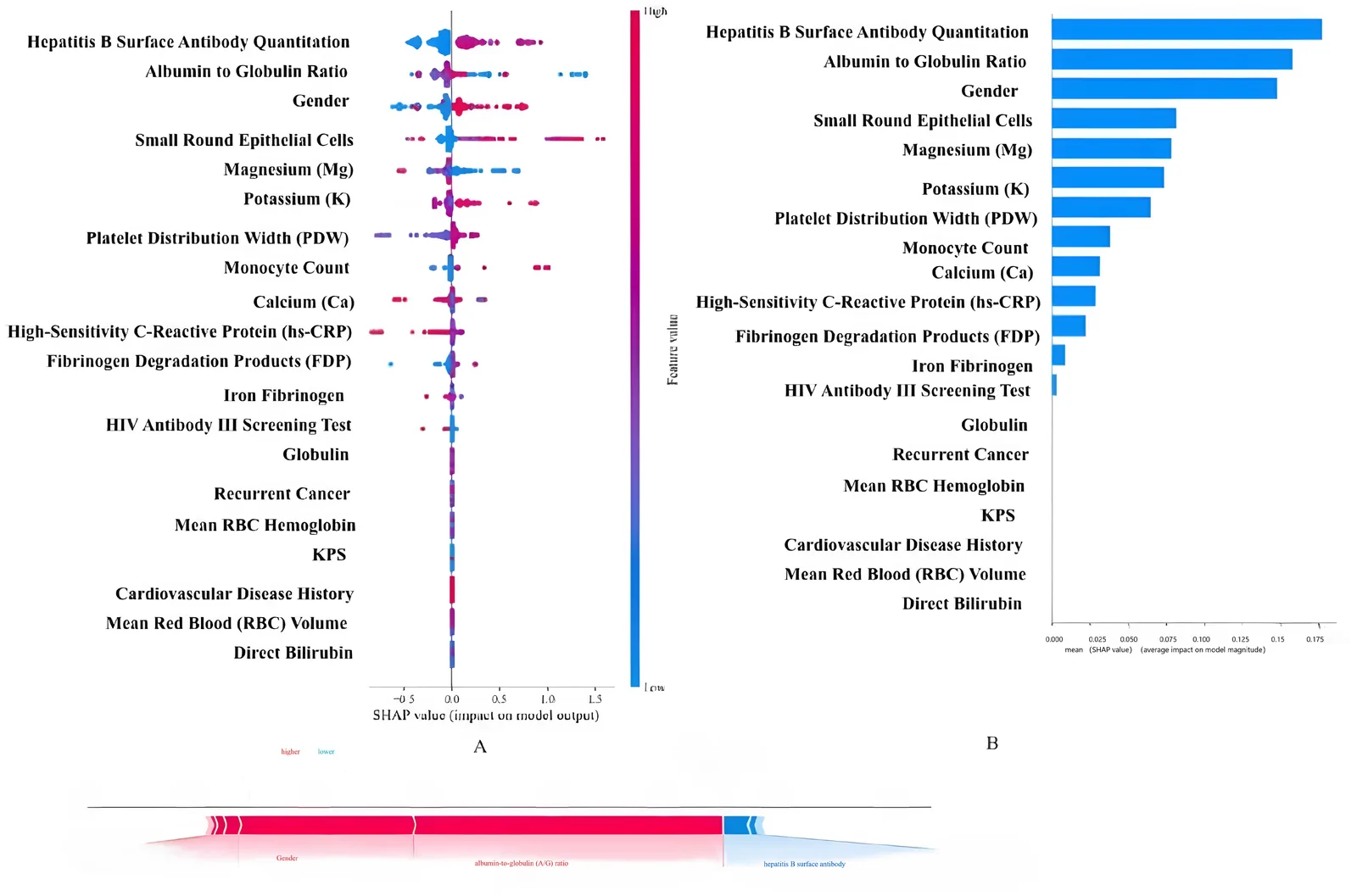
The effects of the various attributes on the model predictions in 1-Year OS predictions with the SHAP method. **(A)** SHAP summary plot. The summary plot combines feature effects and feature importance. The value at each point in the summary plot is the Shapley value for a feature and an instance. **(B)** SHAP feature importance in the form of average absolute SHAP values. **(C)** The mean SHAP values for all samples were obtained, and a force plot was created to illustrate the effect of each feature on average esophageal cancer prognosis.

**Figure 5:**
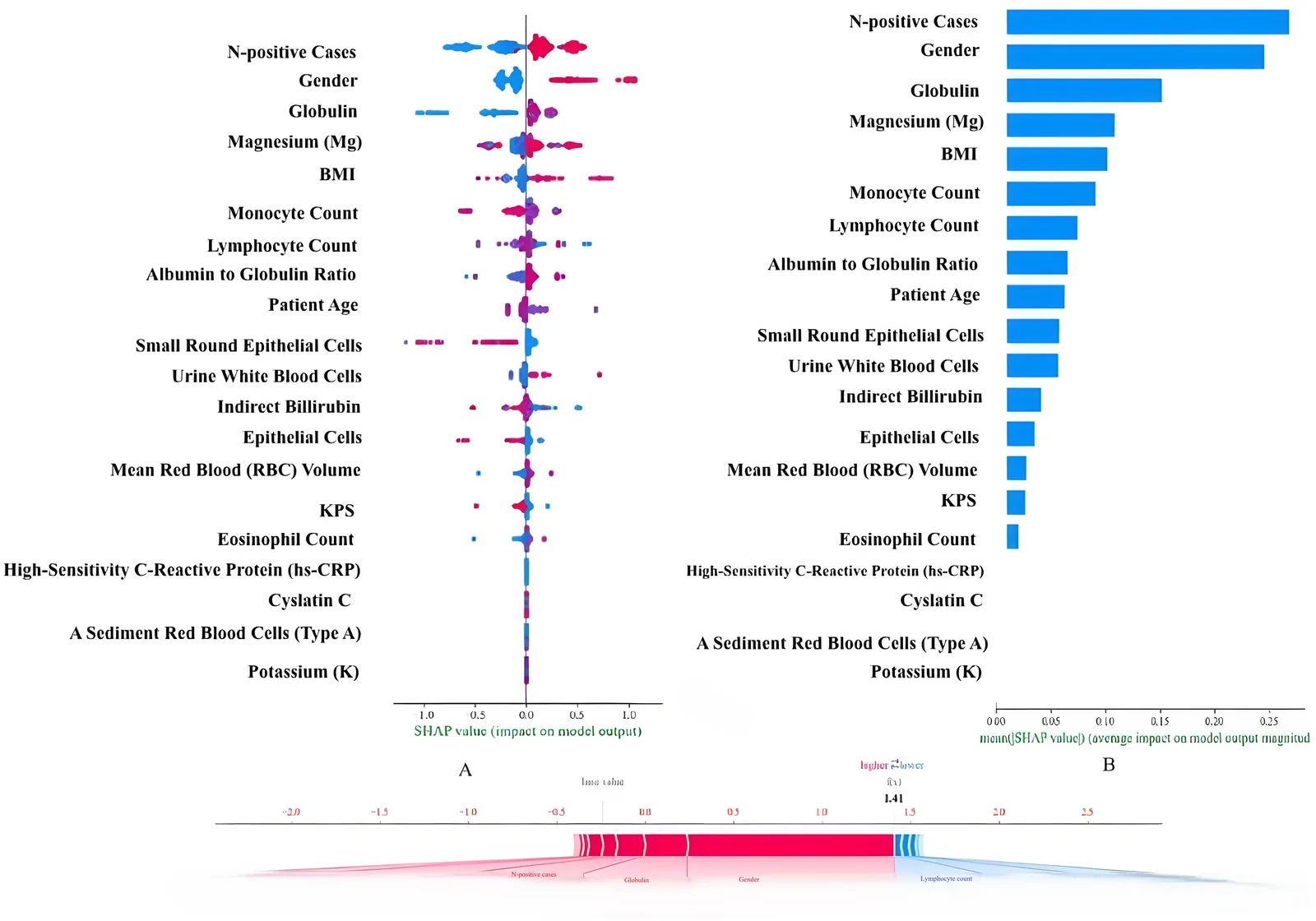
The effect of various features on 5-Year OS predictions using the SHAP method. **(A)** SHAP summary plot. The summary plot combines feature effects and feature importance. All the points on the summary plot are Shapley values of a feature and an instance. The features are ranked by their significance. SHAP feature importance **(B)** SHAPley values are computed as the average of absolute values. The average SHAP values across all samples were computed, and a force plot was created to visually show how each feature affects the average prediction of esophageal cancer prognosis. The effects of increasing risks, like gender, are countered by decreasing effects, like Lymphocyte count. **(C)** The mean SHAP values for all samples were obtained, and a force plot was created to illustrate the effect of each feature on average esophageal cancer prognosis.

Since clinicians are in urgent need of direct, interpretable prediction models, we chose to implement SHAP (Shapley Additive exPlanations). SHAP not only shows the effect of individual features on the model, but also makes the model remarkably easier to understand through its intuitive visualization machinery (see **Figures 4**, **5**). To investigate the interaction between features more deeply, we performed a pairwise analysis of the top 7 features. The interaction highlighted in **Supplementary Figure 7** is that of 1-year predictions, and **Supplementary Figure 8** is the same with 5-year predictions. The SHAP plot of the LightGBM model is displayed in **Supplementary Figure 9**, and the patterns of distributions of important features under various 5-year survival are displayed in **Supplementary Figure 10**. Additional **Supplementary Table 3** identifies the 7 most important features, ranked by SHAP values, in each model and provides clues about their importance.

From a clinical perspective, SHAP-identified variables were interpreted according to their potential actionability. Several influential predictors shown in **Figures 4** and **5**, such as the albumin-to-globulin ratio, monocyte count, lymphocyte count, and electrolyte-related indicators such as serum magnesium, are routinely available before surgery and may support perioperative risk stratification. In contrast, gender and hepatitis B surface antibody quantification should be interpreted mainly as hypothesis-generating findings, because they may reflect complex biological, immunological, or treatment-related associations rather than directly modifiable perioperative targets. Therefore, SHAP outputs were used to improve model interpretability and generate clinically relevant hypotheses, rather than to independently guide treatment decisions.

## 4 Discussion

This article investigated and compared 27 machine learning (ML) models for preoperative baseline examinations in esophageal cancer. From a clinical perspective, the proposed model may provide early prognostic information at the preoperative stage, when treatment planning and perioperative risk management are still being formulated. Because the input variables are routinely collected during standard admission and preoperative evaluation, the model does not impose additional testing burden on patients or clinicians. This may facilitate its integration into electronic medical record systems and support rapid risk stratification before surgery. Patients identified as having a higher predicted risk may benefit from more careful perioperative assessment, individualized treatment planning, and intensified postoperative follow-up. Therefore, the contribution of this study is not limited to algorithmic comparison, but also includes the development of a potentially practical framework for using accessible preoperative non-invasive data in real-world prognostic assessment.

In the past, some progress has been made in the field of esophageal cancer prognosis, but early prognostic evaluation remains tedious, and individual biomarkers have limited predictive capacity (21), making them difficult to apply in practice (22). Comparatively, ML technology is unique in its capacity to handle multidimensional and in-depth data, is flexible in capturing nuanced relationships between variables, and is highly adaptable even in the presence of rapidly changing datasets (23). The tight integration of high-quality, readily available clinical information in electronic medical record (EMR) systems with ML algorithms has certainly provided new opportunities for building clinical prediction models (24). In this work, we made a investigation of models for 1-year and 5-year prognosis prediction. All the performance results are provided in **Supplementary Table 4**. Random Forest (RF) was an excellent model that achieved high scores in various indicators (25). Experimental findings are presented in the **Supplementary Materials**.

In the most important model, we used the SHAP methodology to ensure transparency and interpretability of the designed ML model. Critical factors that determine survival at both 1 and 5 years include gender and blood magnesium concentration. There have been many studies that have indicated that females tend to have a better prognosis. This unequal state may be caused by several factors, including physiological differences, behavioral patterns, and responses to treatment. Indicatively, estrogen is a female sexual hormone that can have inhibitory properties against the growth of esophageal cancerous cells (26). The level of serum magnesium ions can also be an indicator of the prognosis. The poor prognosis of such patients is usually indicated by Hypomagnesemia, where the serum magnesium ion level is low compared to the normal level (27). Magnesium plays a role in the stimulation and functional regulation of immune cells, including T cells (28). Research has always indicated that patients with esophageal cancer and lower concentrations of serum magnesium ions might have accelerated disease development and reduced survival (29). Also, the albumin-to-globulin (A/G) ratio is important in predicting the likelihood of survival at 1 and 5 years after diagnosis.

In determining the prognosis of a patient with esophageal cancer, it is necessary to consider the disease stage, the effectiveness of the treatment administered, the patient’s overall physical condition, and numerous other related factors. The A/G ratio, one of the important indicators of nutritional and immune status, is a predictor of prognosis. In patients with ESCC, a low A/G ratio, an unfavorable prognosis is often anticipated (30). The relationship between a decreased albumin/globulin ratio and poor prognosis in esophageal carcinoma has been supported by numerous studies, making it a robust factor (4). Further, the evidence of the existence of Hepatitis B Virus is a predictive factor of longevity after surgery for this form of cancer in isolation (31). The presence of hepatitis B surface antibody at a high quantitative level can be a good indicator of the good immune functioning of the body. Moreover, considering the survival rate in the case of the five-year period after surgery to cure esophageal cancer, the most prominent factor is the presence or absence of the diagnosis of the invasion of the lymph nodes. The presence or absence of lymph nodes is a significant determinant of the prognosis and overall outcomes of patients because it is directly linked to the disease stage and significantly impacts the decision to provide treatment and prognostic examinations, as shown in the figures below.

The completed ML model is capable of timely delivering clinically significant prognosis assessment data to the patients of esophageal cancer at the first stages of their hospitalization. The model may also provide useful sources of information on how to design their own unique treatment trajectories, despite patients not being qualified to face surgical procedures because of other factors, potential in improving the quality of medical services and patient outcomes (32). Another thing to note is that our model used a flexible selection approach in predicting the first and fifth year survival rates with prognostic factors that are common and distinct as well. Clinically, it is intrinsically difficult to predict 5-year outcomes with only preoperative non-invasive biomarkers, because long-term survival is highly dependent on postoperative pathology, treatment response, and recurrence, among other factors, which cannot be determined prior to the operation. The usage of only preoperative data consequently renders proper 5-year forecasting significantly more challenging and contributes to the explanation of the relatively low performance of the model displayed in our research. Also, our dataset is composed of several thousand patients, and it is significantly larger than the sample sizes employed in different studies.

Not only has this analysis verified the accuracy of the model, but it has also discussed how the predictive information can be successfully incorporated into clinical decision-making processes. By way of example, through knowing the agreement between the risk stratification predicted and the actual patient’s response to the treatment, we have been able to streamline the output of the model, making it more actionable to the clinician. In this effort, we have been able to translate the model’s predictive abilities into a practical change in patient care and outcomes.

The patient cohort that we use in our work is significantly bigger and more varied than in most other previous works, which also complicates but also makes the prediction task more realistic, reflecting actual clinical circumstances. Besides, the objective is not to maximize predictive accuracy, but to demonstrate that early prognosis can be implemented with the help of preoperative non-invasive biomarker. To reduce the popular critique of the ML models as black boxes, we used the SHAP method to eliminate the constraint inherent in the black box models. It is a model prediction technique that elucidates model predictions, in addition to offering intuitive and detailed prediction bases to clinicians in terms of a combination of global and local explanations, thus increasing the acceptability and clinical viability of the model.

Although the predictive performance is promising, as it has been illustrated in our study, there are nonetheless some limitations. Even though the dataset is rather large and consists of preoperative non-invasive biomarkers in their entirety, and with long-term survival follow-up, single-center data might be unable to fully describe the level of heterogeneity in population characteristics across various institutions. As a result, it is possible that the generalizability and strength of our model can be reduced when it is used in wider clinical conditions. Secondly, the sample of the data used may not be a true representative of the overall population of patients with esophageal carcinoma, and the model may not be as precise across different groups of patients. Moreover, the research also targeted a particular range of clinical and laboratory manifestations, and other possible significant predictors might have been missed.

## 5 Conclusion

In this study, we developed an interpretable machine learning framework based on routinely available preoperative electronic medical record (EMR) data. The framework was designed for exploratory early risk stratification in patients with esophageal squamous cell carcinoma. Among the evaluated algorithms, the Random Forest model demonstrated the best overall predictive performance. SHAP analysis further provided clinically meaningful insights into the model’s predictions. The proposed framework may facilitate the early identification of patients at different levels of postoperative risk. It may also provide additional evidence to support individualized perioperative management. However, this study was based on a retrospective single-center cohort. Therefore, the proposed model should be regarded as an exploratory risk-stratification tool rather than a clinically mature prediction system. Further multicenter external validation and prospective studies are needed before routine clinical application. Future work will focus on validating the framework in larger, multicenter, and more heterogeneous cohorts. We also plan to incorporate additional clinically relevant predictors. In addition, we will explore advanced artificial intelligence approaches, including deep learning and large language models, to further improve predictive performance, robustness, and generalizability.

## Data Availability

The raw data supporting the conclusions of this article will be made available by the authors, without undue reservation.
