# Supplementary material for: Building an interpretable machine learning prognosis prediction model-based on baseline examinations of patients with esophageal cancer undergoing surgery
Source: Front Oncol. 2026 Aug 14;16:1839382. doi: 10.3389/fonc.2026.1839382 (PMC13521834; doi:10.3389/fonc.2026.1839382)
Supplement: Supplementary file 1 [file Table1.docx]

Supplementary Material

# 1 Supplementary Figures

**Supplementary Figure S1.** Correlation map of selected features for 1-year prediction.

**Supplementary Figure S2.** Correlation map of selected features for 5-year prediction.

**Supplementary Figure S3.** Model performance regarding the AUC of 1-year and 5-year prediction.

**Supplementary Figure S4.** Model performance regarding the F1score of 1-year and 5-year survival predictions.

**Supplementary Figure S5.** Confusion matrix results for 1-year and 5-year survival predictions of the RF.

**Supplementary Figure S6.** AUC Values Obtained from Training the Optimized RF Model.

**Supplementary Figure S7.** Interaction Between Different Features in 1-Year Predictions The horizontal and vertical coordinate labels are consistent, representing pairwise comparisons of interactions among the top 7 features.

**Supplementary Figure S8.** Interaction Between Different Features in 5-Year Predictions The horizontal and vertical coordinate labels are consistent, representing pairwise comparisons of interactions among the top 7 features.

**Supplementary** **Figure S9.** The SHAP plot of LightGBM.

**Supplementary** **Figure S10.** The distribution patterns of key relevant features across various 5-year survival periods.

**Supplementary** **Figure S11.** The ROC curve of the Random Forest (RF) model for predicting 1-year overall survival (OS). The blue solid line represents the RF model, and the diagonal grey dashed line represents a random classifier.

**Supplementary** **Figure S12.** Decision Curve Analysis (DCA) of the Random Forest (RF) model for 1-year overall survival (OS).

**Supplementary** **Figure S13.** The ROC curve of the Random Forest (RF) model for predicting 5-year overall survival (OS).

**Supplementary** **Figure S14.** Decision Curve Analysis (DCA) of the Random Forest (RF) model for 5-year overall survival (OS).

# 2 Tables

**Supplementary Table S1 (a). Baseline clinical characteristics and survival outcome distribution of the study cohort。**

| Gender | Male | 1946 |
| --- | --- | --- |
|  | Female | 406 |
| KPS | 2 | 1420 |
|  | 3 | 912 |
|  | 4 | 20 |
| Cardiovascular Disease History | No | 2226 |
|  | Yes | 126 |
| Recurrent Cancer | NO | 2290 |
|  | Yes | 62 |
| N-positive Cases | Yes | 1379 |
|  | NO | 971 |
| 1yearOS | 1 (Dead) | 556 |
|  | 0 (Alive) | 1796 |
| 5yearOS | 1 (Dead) | 1553 |
|  | 0 (Alive) | 799 |

**Supplementary Table S1 (b): Details of the baseline features included in the study. This table summarizes all preoperative non-invasive biomarkers and clinical examination indicators used as input features for model development.**

|  | **Mean** | **Median** | **Standard deviation** |
| --- | --- | --- | --- |
| Patient Age | 60.95 | 61 | 8.281 |
| BMI | 20.22 | 19.68 | 18.57 |
| Mean Red Blood Cell (RBC) Volume | 95.169 | 95.6 | 7.95 |
| Mean RBC Hemoglobin | 31.32 | 31.4 | 3.917 |
| Mean RBC Hemoglobin Concentration | 326.527 | 326 | 11.36 |
| Platelet Distribution Width (PDW) | 16.428 | 16.4 | 0.6161 |
| WBC | 6.37 | 6.05 | 1.975 |
| Lymphocyte Count | 1.56679 | 1.51 | 0.5346 |
| Monocyte Count | 0.4123 | 0.38 | 0.17854 |
| Eosinophil Count | 0.1768 | 0.13 | 0.1818 |
| Basophil Count | 0.02219 | 0.02 | 0.022415 |
| Reticulocyte Count | 0.04328 | 0.0397 | 0.0197 |
| High-Sensitivity C-Reactive Protein (hs-CRP) | 11.80811 | 4.496 | 21.432 |
| Direct Bilirubin | 5.17224 | 4.7 | 2.586 |
| Indirect Bilirubin | 8.696 | 8.2 | 3.9778 |
| Total Protein | 73.451 | 70.6 | 142.78 |
| Globulin | 27.816 | 27.5 | 4.46 |
| Albumin to Globulin Ratio | 1.567 | 1.551 | 0.27 |
| Creatinine | 73.353 | 72.5 | 14.806 |
| Triglycerides | 1.3 | 1.11 | 0.885 |
| Potassium (K) | 4.386 | 4.35 | 0.459 |
| Sodium (Na) | 140.524 | 140.7 | 2.834 |
| Chloride (Cl) | 103.94 | 104 | 3.246 |
| Calcium (Ca) | 2.406 | 2.4 | 0.147 |
| Magnesium (Mg) | 0.9385 | 0.94 | 0.103 |
| Phosphorus (P) | 1.114 | 1.11 | 0.203 |
| Cystatin C | 0.992 | 0.935 | 0.506 |
| Total Bile Acid | 6.814 | 4.3 | 9.29 |
| Lipase | 35.4 | 32.34 | 16.6 |
| X5 Nucleotidase | 5.893 | 4.57 | 9.56 |
| Iron | 16.34 | 13 | 14.87 |
| Total Carbon Dioxide | 25.653 | 25.5 | 3.117 |
| Prothrombin Time (PT) | 14.697 | 15.6 | 3.156 |
| International Normalized Ratio (INR) | 0.977 | 0.93 | 1.565 |
| Fibrinogen | 3.344 | 3.24 | 0.904 |
| Thrombin Time (TT) | 15.342 | 16 | 2.799 |
| D-Dimer | 3.1723 | 0.31 | 16.066 |
| Fibrinogen Degradation Products (FDP) | 3.051 | 1.5 | 9.743 |
| Hepatitis B Surface Antibody Quantitation | 115.54 | 24.43 | 217.38 |
| Hepatitis B e Antibody Quantitation | 0.743 | 0.159 | 1.394 |
| Hepatitis C Antibody | 0.251 | 0.129 | 1.277 |
| HIV Antibody III Screening Test | 0.051 | 0.007 | 0.0972 |
| Treponema Pallidum Antibody | 0.1406 | 0.1071 | 0.1378 |
| Number of Crystals | 11.687 | 0.1 | 60.82 |
| Urine White Blood Cells | 25.135 | 4.4 | 129.668 |
| Epithelial Cells | 5.492 | 2.6 | 9.55 |
| Small Round Epithelial Cells | 2.416 | 1.1 | 4.306 |
| Pathological Casts | 0.36 | 0.12 | 1.07 |
| Sediment Specific Gravity | 1.02 | 1.02 | 0.004 |
| Sediment Casts | 1.332 | 0.26 | 6.888 |
| Sediment Bacteria | 590.47 | 41.2 | 4399.93 |
| A Sediment Red Blood Cells (Type A) | 53.27 | 5.6 | 770.17 |

**Supplementary Table S2**

| **Model** | **Hyperparameters** | **Value** |
| --- | --- | --- |
|  | base_estimator | DecisionTreeClassifier(max_depth=1) |
| AdaBoostClassifier | n_estimators | 50 |
|  | base_estimator | DecisionTreeClassifier |
| BaggingClassifier | n_estimators | 10 |
|  | alpha | 1 |
| BernoulliNB | binarize | 0 |
|  | base_estimator | None |
| CalibratedClassifierCV | method | sigmoid |
|  | min_samples_split | 2 |
| DecisionTreeClassifier | min_samples_leaf | 1 |
|  | strategy | prior |
| DummyClassifier | random_state | None |
|  | min_samples_split | 2 |
| ExtraTreeClassifier | min_samples_leaf | 1 |
|  | n_estimators | 100 |
| ExtraTreesClassifier | criterion | gini |
|  | priors | None |
| GaussianNB | var_smoothing | 0.000000001 |
|  | n_neighbors | 5 |
| KNeighborsClassifier | weights | uniform |
|  | kernel | rbf |
| LabelPropagation | gamma | 20 |
|  | kernel | rbf |
| LabelSpreading | gamma | 20 |
|  | solver | svd |
| LinearDiscriminantAnalysis | shrinkage | None |
|  | penalty | l2 |
| LinearSVC | loss | squared_hinge |
|  | penalty | l2 |
| LogisticRegression | dual | FALSE |
|  | metric | euclidean |
| NearestCentroid | shrink_threshold | None |
|  | nu | 0.5 |
| NuSVC | kernel | rbf |
|  | C | 1 |
| PassiveAggressiveClassifier | fit_intercept | TRUE |
|  | penalty | None |
| Perceptron | alpha | 0.0001 |
|  | priors | None |
| QuadraticDiscriminantAnalysis | reg_param | 0 |
|  | n_estimators | 10 |
| RandomForestClassifier | criterion | gini |
|  | alpha | 1 |
| RidgeClassifier | fit_intercept | TRUE |
|  | loss | hinge |
| SGDClassifier | penalty | l2 |
|  | C | 1 |
| SVC | kernel | rbf |
|  | booster | gbtree |
| XGBClassifier | n_estimators | 100 |
|  | boosting_type | gbdt |
| LGBMClassifier | num_leaves | 31 |

**Supplementary Table S3. Top 7 features ranked by SHAP values for different models.**

| **RF (1 year)** | **lightGBM (1year)** | **RF (5 year)** | **lightGBM (5 year)** |
| --- | --- | --- | --- |
| Hepatitis B surface Antibody Quantitation | Hepatitis B surface Antibody Quantitation | N-positive Cases | N-positive Cases |
| Albumin to Globulin ratio | hs-CRP | Gender | Mean Red Blood Cell Volume |
| Gender | FDP | Globulin | Albumin to Globulin ratio |
| Small round Epithelial Cells | Monocyte Count | Magnesium（Mg） | Potassium |
| Magnesium（Mg） | Calcium | BMI | BMI |
| Potassium | Small round Epithelial Cells | Monocyte Count | Monocyte Count |
| Platelet Distribution width(PDW) | Gender | Lymphocyte Count | Cystatin C |

**Supplementary Table S4. Machine learning algorithms experiment results using SMOTE data augmentation techniques using 10-fold cross validation.**

|  | **AdaBoost** | **BaggingClassifier** | **BernoulliNB** | **CalibratedClassifier** | **DecisionTree** | **DummyClassifier** | **ExtraTree Classifier** | **ExtraTrees Classifier** | **GaussianNB** | **KNeighbors** | **LabelPropagation** |
| --- | --- | --- | --- | --- | --- | --- | --- | --- | --- | --- | --- |
| 1 year Accuracy (mean) | 0.66 | 0.66 | 0.66 | 0.66 | 0.66 | 0.66 | 0.66 | 0.66 | 0.66 | 0.66 | 0.66 |
| 1 year ROC AUC  (mean) | 0.58 | 0.58 | 0.58 | 0.58 | 0.58 | 0.58 | 0.58 | 0.58 | 0.58 | 0.58 | 0.58 |
| 1 year F1 Score  (mean) | 0.67 | 0.67 | 0.67 | 0.67 | 0.67 | 0.67 | 0.67 | 0.67 | 0.67 | 0.67 | 0.67 |
| 5 year Accuracy (mean) | 0.63 | 0.62 | 0.59 | 0.64 | 0.59 | 0.34 | 0.58 | 0.66 | 0.43 | 0.56 | 0.55 |
| 5 year ROC AUC  (mean) | 0.62 | 0.61 | 0.62 | 0.64 | 0.57 | 0.50 | 0.55 | 0.63 | 0.56 | 0.59 | 0.55 |
| 5 year F1 Score  (mean) | 0.64 | 0.62 | 0.60 | 0.65 | 0.59 | 0.17 | 0.58 | 0.66 | 0.36 | 0.57 | 0.57 |

**Supplementary Table S4. Machine learning algorithms experiment results using SMOTE data augmentation techniques using 10-fold cross validation (continue).**

|  | **LabelSpreading** | **LinearDiscriminantAnalysis** | **LinearSVC** | **LogisticRegression** | **NearestCentroid** | **NuSVC** | **PassiveAggressiveClassifier** | **Perceptron** | **QuadraticDiscriminantAnalysis** | **RandomForestClassifier** | **RidgeClassifier** |
| --- | --- | --- | --- | --- | --- | --- | --- | --- | --- | --- | --- |
| 1 year Accuracy (mean) | 0.60 | 0.64 | 0.64 | 0.64 | 0.68 | 0.65 | 0.57 | 0.56 | 0.74 | **0.74** | 0.64 |
| 1 year ROC AUC  (mean) | 0.53 | 0.63 | 0.63 | 0.63 | 0.64 | 0.54 | 0.55 | 0.55 | 0.59 | **0.69** | 0.63 |
| 1 year F1 Score  (mean) | 0.62 | 0.66 | 0.67 | 0.66 | 0.70 | 0.65 | 0.60 | 0.59 | 0.72 | **0.72** | 0.66 |
| 5 year Accuracy (mean) | 0.55 | 0.64 | 0.64 | 0.64 | 0.60 | 0.63 | 0.53 | 0.56 | 0.40 | **0.67** | 0.64 |
| 5 year ROC AUC  (mean) | 0.55 | 0.64 | 0.64 | 0.64 | 0.62 | 0.61 | 0.54 | 0.56 | 0.54 | **0.62** | 0.64 |
| 5 year F1 Score  (mean) | 0.57 | 0.65 | 0.65 | 0.65 | 0.61 | 0.63 | 0.54 | 0.57 | 0.29 | **0.67** | 0.65 |

**Supplementary Table S4. Machine learning algorithms experiment results using SMOTE data augmentation techniques using 10-fold cross validation (continue).**

|  | **RidgeClassifierCV** | **SGDClassifier** | **SVC** | **XGBClassifier** | **LGBMClassifier** |
| --- | --- | --- | --- | --- | --- |
| 1 year Accuracy (mean) | 0.64 | 0.60 | 0.65 | 0.72 | 0.72 |
| 1 year ROC AUC  (mean) | 0.63 | 0.58 | 0.56 | 0.57 | 0.56 |
| 1 year F1 Score  (mean) | 0.66 | 0.63 | 0.66 | 0.70 | 0.70 |
| 5 year Accuracy (mean) | 0.64 | 0.58 | 0.64 | 0.65 | 0.67 |
| 5 year ROC AUC  (mean) | 0..64 | 0.60 | 0.63 | 0.60 | 0.62 |
| 5 year F1 Score  (mean) | 0.65 | 0.59 | 0.65 | 0.65 | 0.66 |
